# Tuberculosis and viral hepatitis in patients treated with certolizumab pegol in Asia-Pacific countries and worldwide: real-world and clinical trial data

**DOI:** 10.1007/s10067-020-05248-4

**Published:** 2020-08-01

**Authors:** Chak Sing Lau, Yi-Hsing Chen, Keith Lim, Marc de Longueville, Catherine Arendt, Kevin Winthrop

**Affiliations:** 1 Department of Medicine, Queen Mary Hospital, University of Hong Kong, Pok Fu Lam, Hong Kong; 2 Taichung Veterans General Hospital, Taichung, Taiwan; 3 Western Health Rheumatology Unit and AIMSS, Melbourne University, Melbourne, Australia; 4 UCB Pharma, Brussels, Belgium; 5 Oregon Health and Science University, Portland, OR USA

**Keywords:** Pharmacovigilance, Psoriatic arthritis, Rheumatic diseases, Rheumatoid arthritis, Spondyloarthritis

## Abstract

**Introduction/objectives:**

To evaluate the incidence rate (IR) of tuberculosis (TB) and viral hepatitis B and C (HBV/HCV) during certolizumab pegol (CZP) treatment, worldwide and in Asia-Pacific countries, across clinical trials and post-marketing reports (non-interventional studies and real-world practice).

**Method:**

CZP safety data were pooled across 49 clinical trials from 1998 to June 2017. Post-marketing reports were from initial commercialization until March 2015 (TB)/February 2017 (HBV/HCV). All suspected TB and HBV/HCV cases underwent centralized retrospective review by external experts. Incidence rates (IRs) were calculated per 100 patient-years (PY) of CZP exposure.

**Results:**

Among 11,317 clinical trial patients (21,695 PY), 62 TB cases were confirmed (IR 0.29/100 PY) including 2 in Japan (0.10/100 PY) and 3 in other Asia-Pacific countries (0.58/100 PY). From > 238,000 PY estimated post-marketing CZP exposure, there were 31 confirmed TB cases (0.01/100 PY): 5 in Japan (0.05/100 PY), 1 in other Asia-Pacific countries (0.03/100 PY). Reported regional TB IRs were highest in eastern Europe (0.17/100 PY), central Europe (0.09/100 PY), and Mexico (0.16/100 PY). Across clinical trials, there was 1 confirmed HBV reactivation and no HCV cases. From > 420,000 PY estimated post-marketing CZP exposure, 5 HBV/HCV cases were confirmed (0.001/100 PY): 2 HCV reactivations; 1 new HCV; plus 2 HBV reactivations in Japan (0.008/100 PY).

**Conclusions:**

CZP TB risk is aligned with nationwide TB rates, being slightly higher in Asia-Pacific countries excluding Japan. Overall, TB and HBV/HCV risk with CZP treatment is currently relatively low, as risk can be minimized with patient/physician education, screening, and vigilant treatment, according to international guidelines.

**Table:** 

**Key Points:** *• TB rates were highest in eastern/central Europe, Mexico, and Asia-Pacific regions.* *• With the implementation of stricter TB screening and risk evaluations in 2007, especially in high TB incidence countries, there was a notable reduction TB occurrence.* *• Safety profile of biologics in real-world settings complements controlled studies.* *• TB and hepatitis (HBV/HCV) risk with certolizumab pegol (CZP) treatment is low.*

**Electronic supplementary material:**

The online version of this article (10.1007/s10067-020-05248-4) contains supplementary material, which is available to authorized users.

## Introduction

Patients with immune-mediated inflammatory diseases (IMIDs) have a greater risk of serious infectious events (SIEs) than the general population [1]. The use of biologics and anti-tumor necrosis factor drugs (anti-TNF) is associated with an increased risk of infections including tuberculosis (TB) [2–5], and possibly viral hepatitis B (HBV) and viral hepatitis C (HCV) [6–8]. This is a particularly relevant clinical consideration in the Asia-Pacific region, where nationwide incidence rates (IR) of TB and HBV/HCV are relatively high [9–11]. An estimated 45% of global TB cases in 2016 occurred in Southeast Asia [11], and TB is among the commonest causes of death in this region [12]. In Japan and other countries, TB control has improved dramatically over the past few decades [13], and in the World Health Organization (WHO) Western Pacific region, like the rest of the world, TB mortality in the HIV-negative population has been steadily decreasing [11]. However, Japan remains a moderate TB burden country with an incidence estimate of 15 cases per 100,000 population in 2017 [14], most of which are diagnosed in patients aged over 75 years [15].

In higher TB and HBV/HCV incidence areas, global and regional safety data can support education of local physicians, and decision-making around anti-TNF treatment in the context of optimal screening and diagnosis procedures. Certolizumab pegol (CZP) is an Fc-free, PEGylated anti-TNF, approved for the treatment of adult patients with rheumatoid arthritis (RA), axial spondyloarthritis (axSpA; including ankylosing spondylitis [AS] and non-radiographic axSpA), psoriatic arthritis (PsA), plaque psoriasis (PSO), and Crohn’s disease (CD) [16, 17], although registered indications vary by country. In Asia-Pacific countries, CZP is currently approved for the treatment of RA, AS/axSpA, PsA, and PSO.

This paper reports IR of TB, HBV, and HCV during CZP treatment in pooled data from clinical trials of CZP across its approved indications, as well as post-marketing reports (non-interventional studies [NIS] and real-world clinical practice), both worldwide and in the Asia-Pacific region.

## Materials and methods

### Data sources and patient populations

**Fig. 1:**
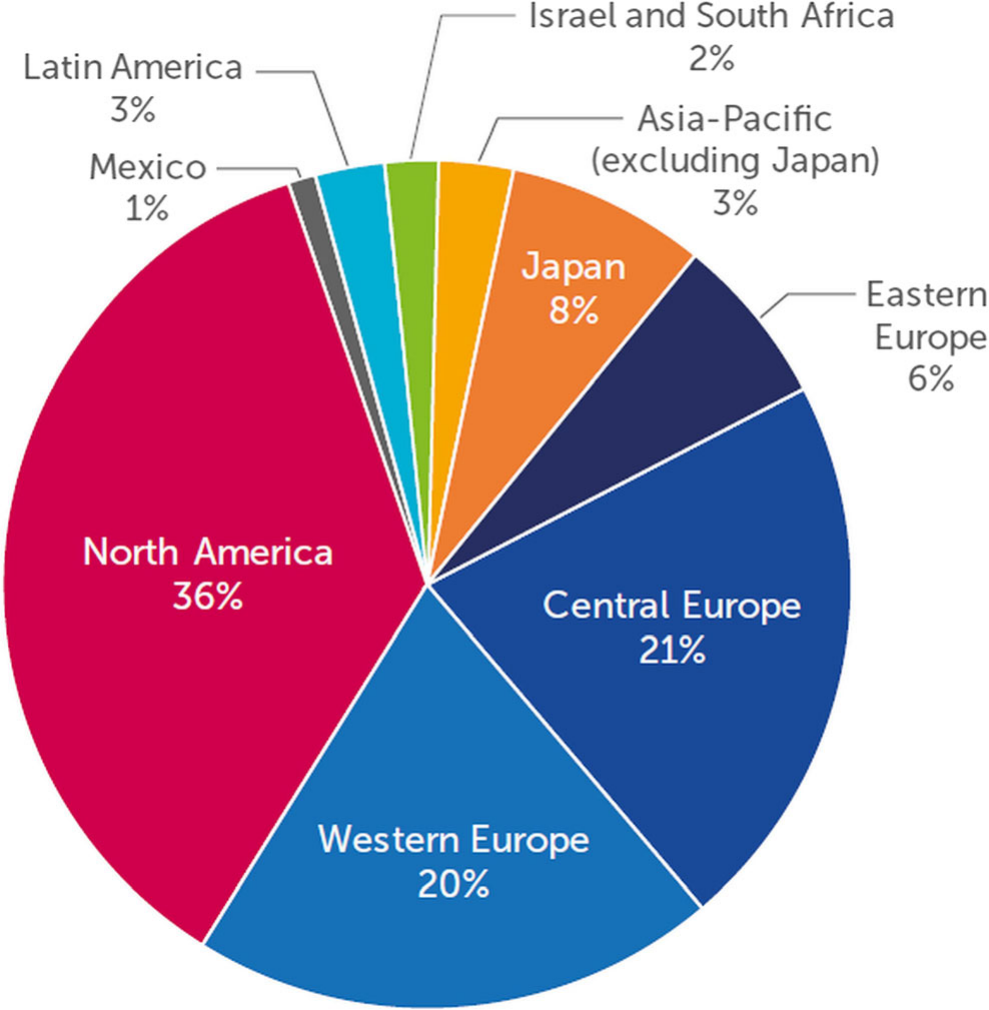
Geographic distribution of patients included in CZP clinical trials Asia-Pacific: Australia, Hong Kong, New Zealand, Republic of Korea, Singapore; eastern Europe: Belarus, Croatia, Georgia, Romania, Russia, Serbia, Ukraine; central Europe: Bulgaria, Czech Republic, Estonia, Hungary, Latvia, Lithuania, Poland, Slovakia, Slovenia; western Europe: Austria, Belgium, Denmark, Finland, France, Germany, Greece, Ireland, Italy, The Netherlands, Norway, Portugal, Spain, Sweden, Switzerland, UK; North America: Canada, US; Latin America: Argentina, Brazil, Chile, Colombia; Rest of the World: Israel, South Africa. CZP, certolizumab pegol

CZP safety data were pooled across 49 clinical trials (27 in RA, 15 in CD, 1 in axSpA, 1 in PsA, and 5 in PSO), including open label extensions (OLEs). The latest data cut was 30 June 2017, for PSO. Clinical trials were performed across a total of 45 countries (Fig. 1), including substantial numbers of patients from countries with a higher nationwide TB incidence, such as Ukraine (87/100,000), Russia (66/100,000), Republic of Korea (77/100,000), Singapore (51/100,000), and South Africa (781/100,000) [11]. Asia-Pacific countries included Australia, Hong Kong, Republic of Korea, New Zealand, and Singapore (Supplementary Table S1). As Japan represented the majority of real-world CZP exposure in the Asia-Pacific region, data were reported separately.

CZP dosing during interventional studies varied depending on the indication, study, and treatment arm, with patients exposed to 200 mg every 2 weeks (Q2W), 400 mg every 4 weeks (Q4W), or CZP 400 mg Q2W. A 400-mg loading dose (LD) at weeks 0, 2, and 4 was used across most studies and indications [18].

Post-marketing data (mostly RA and CD) represent all NIS and spontaneous adverse event (AE) reports recorded worldwide in the sponsor pharmacovigilance database since CZP became commercially available in 2008 (to treat CD, USA [16]). Asia-Pacific countries included in the post-marketing data were Australia, Hong Kong, Republic of Korea, Malaysia, New Caledonia, and Japan (Supplementary Table S1).

### Safety assessments

Clinical trial safety data included all TB and HBV/HCV events from first CZP dose to 70 days after the last dose (5 times the half-life of CZP) or withdrawal. Suspected cases of TB disease in this paper were reported using the WHO or Centers for Disease Control and Prevention (CDC) TB criteria [3, 19], before undergoing centralized external expert review. Investigators were required to systematically report cases of TB disease as serious adverse events (SAEs).

In CZP clinical trials before 2007, as with other biologics, TB screening was conducted according to local guidelines. However, an increased risk of TB was observed during the clinical development of infliximab and adalimumab [20], and also of CZP in the USA and Europe [21, 22]. As a result, from 2007, stricter screening rules were applied (Supplementary Information).

The sponsor CZP pharmacovigilance database provides (S) AE reports from a range of worldwide sources (including spontaneous reports from healthcare professionals and consumers, NIS, and registries), as well as SAE reports from clinical trials of any sponsor compounds in development. AE information available includes patients’ age, sex, country, and a clinical narrative including follow-up to event resolution. For this study, post-marketing data included all TB and HBV/HCV cases reported in the sponsor pharmacovigilance database to March 6, 2015, for TB (collated for a health regulatory authority request), and February 27, 2017, for HBV/HCV. MedDRA (version 18.1) preferred terms used to identify potential post-marketing cases of TB, HBV, and HCV in the sponsor pharmacovigilance database are listed in Supplementary Table S2. Time to event categories (0–< 3, 3–< 6, 6–< 12, 12–< 24, 24–< 36 months) for post-marketing TB events was determined by sponsor medical review, due to the variability of information recorded in the pharmacovigilance narratives.

### External medical review of suspected TB, HBV, and HCV cases

A retrospective external review of suspected cases of TB, HBV, and HCV from both CZP clinical trials and post-marketing data was conducted by a committee of independent experts (C-S. Lau, Y-H. Chen, K. Lim, K. Winthrop; plus X. Mariette for clinical trial TB cases only). As well as the verbatim and preferred term, information from the Council for International Organizations of Medical Sciences (CIOMS) form and/or narrative in the sponsor pharmacovigilance database were used where available.

All suspected TB cases from clinical trials and post-marketing data were reviewed and classified as “Confirmed” (case met the TB disease clinical case definition or TB disease laboratory criteria, as defined by the WHO and CDC [19]), “Doubtful” (incomplete or contradictory evidence), “Rejected” (WHO/CDC criteria were clearly not met), or “Unassessable” (not enough information available), using the methodology described previously [3]. Confirmed TB cases were further categorized by site of infection as “Pulmonary,” “Non-pulmonary,” “Disseminated,” or “Unassessable.” LTBI cases were excluded.

All suspected cases of HBV and HCV were also expert-reviewed, and classified as “Confirmed,” “Doubtful,” “Rejected,” or “Unassessable” according to CDC criteria for the interpretation of HBV [23] and HCV [24] test results. Where HBV DNA tests had been completed and returned positive, patients were classified as confirmed active cases of HBV. Each confirmed case of HBV or HCV was also classified as new or reactivation of a previous HBV/HCV infection.

### Statistical analysis

Statistical analysis used the SAS® software version 9.4 (SAS Institute Inc., Cary, NC, USA). IR per 100 patient-years (PY) were calculated from total CZP exposure with 95% confidence intervals (CIs). The cumulative post-marketing exposure of CZP in PYs for each country was estimated based on commercial sales:

$$ \mathrm{Patient}\hbox{-} \mathrm{years}\ \left(\mathrm{PY}\right)=\frac{\mathrm{total}\ \mathrm{mg}\ \mathrm{of}\ \mathrm{product}\ \mathrm{distributed}/\mathrm{monthly}\ \mathrm{dose}}{12} $$

Actual monthly dose was assumed as 400 mg according to the approved dosing regimen of CZP 200 mg Q2W or CZP 400 mg Q4W. This did not include correction for the prescribed LD of CZP 400 mg at weeks 0, 2, and 4 of treatment.

### Statement of human and animal rights

All clinical trials complied with the Declaration of Helsinki.

## Results

### Patient population

**Fig. 2:**
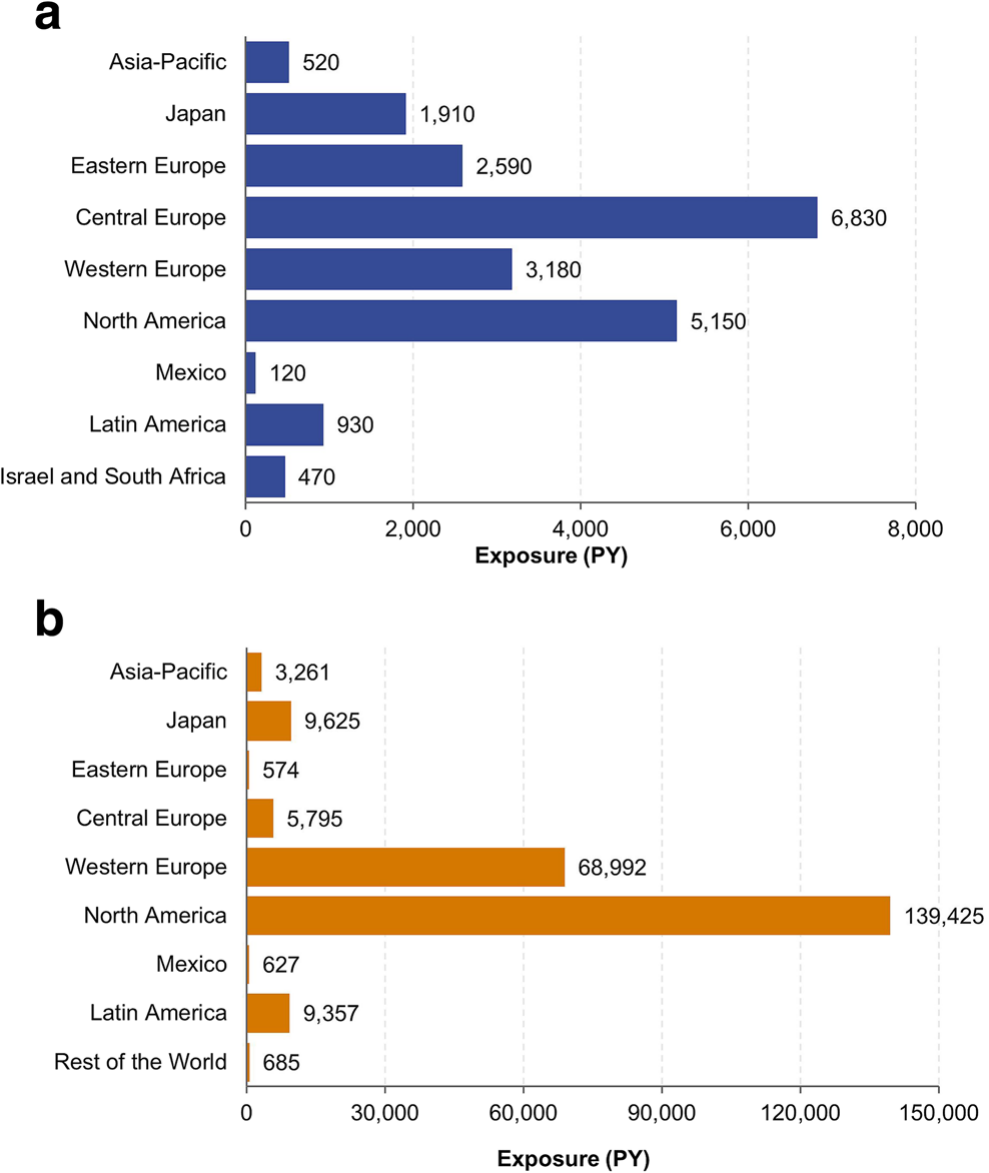
Cumulative exposure to CZP per region or country in clinical trial and post-marketing TB analysis. **a** Pooled clinical trial CZP exposure Asia-Pacific: Australia, Hong Kong, New Zealand, Republic of Korea, Singapore; eastern Europe: Belarus, Croatia, Georgia, Romania, Russia, Serbia, Ukraine; central Europe: Bulgaria, Czech Republic, Estonia, Hungary, Latvia, Lithuania, Poland, Slovakia, Slovenia; western Europe: Austria, Belgium, Denmark, Finland, France, Germany, Greece, Ireland, Italy, The Netherlands, Norway, Portugal, Spain, Sweden, Switzerland, UK; North America: Canada, US; Latin America: Argentina, Brazil, Chile, Colombia. CZP, certolizumab pegol; PY, patient-years; TB: tuberculosis. **b** Post-marketing CZP exposure to 6 March 2015 (TB cut-off date) Asia-Pacific: Australia, Hong Kong, Malaysia, New Caledonia, Republic of Korea; eastern Europe: Romania, Russia; central Europe: Bulgaria, Czech Republic, Hungary, Poland, Slovakia, Slovenia; western Europe: Andorra, Austria, Belgium, Denmark, Finland, France, Germany, Greece, Ireland, Italy, The Netherlands, Norway, Portugal, Spain, Sweden, Switzerland, UK; North America: Canada, US; Latin America: Argentina, Brazil, Chile, Colombia, Ecuador, French Guiana, Guadeloupe, Peru, Reunion; rest of the world: Bahrain, Cyprus, Egypt, Kuwait, Lebanon, Libya, Martinique, Qatar, Saudi Arabia, Turkey, UAE. CZP, certolizumab pegol; PY, patient-years; TB, tuberculosis

Across clinical trials, CZP was administered globally to 11,317 patients (total exposure of 21,695 PY): 6927 patients with RA (13,542 PY), 2570 patients with CD (4378 PY), 315 patients with axSpA (978 PY), 393 patients with PsA (1316 PY), and 1112 patients with PSO (1481 PY). A total of 4602 patients (41%) commenced CZP treatment prior to 2007; 6715 patients (59%) commenced CZP treatment in 2007 or later. Mean duration of CZP exposure per patient was 1.9 years (median 1.2 years); maximum individual exposure was 7.8 years. Further baseline characteristics for this patient population have been reported previously [18]. Central Europe was the geographic region with the highest CZP exposure across clinical trials (6830 PY). CZP exposure totaled 520 PY in the Asia-Pacific region (excluding Japan), and 1910 PY in Japan (Fig. 2a).

Total post-marketing exposure to CZP was estimated at > 238,000 PY for TB data (up to March 6, 2015) and > 420,000 PY for HBV/HCV data (up to February 27, 2017). North America had the highest estimated total post-marketing CZP exposure (> 139,000 PY for the TB dataset, including RA, axSpA, PsA, and CD), followed by western Europe (> 68,000 PY) and Japan (10,000 PY) (both rheumatological indications only). In the Asia-Pacific region excluding Japan, total CZP exposure was > 3000 PY (Fig. 2b).

### TB incidence across clinical trials

**Fig. 3:**
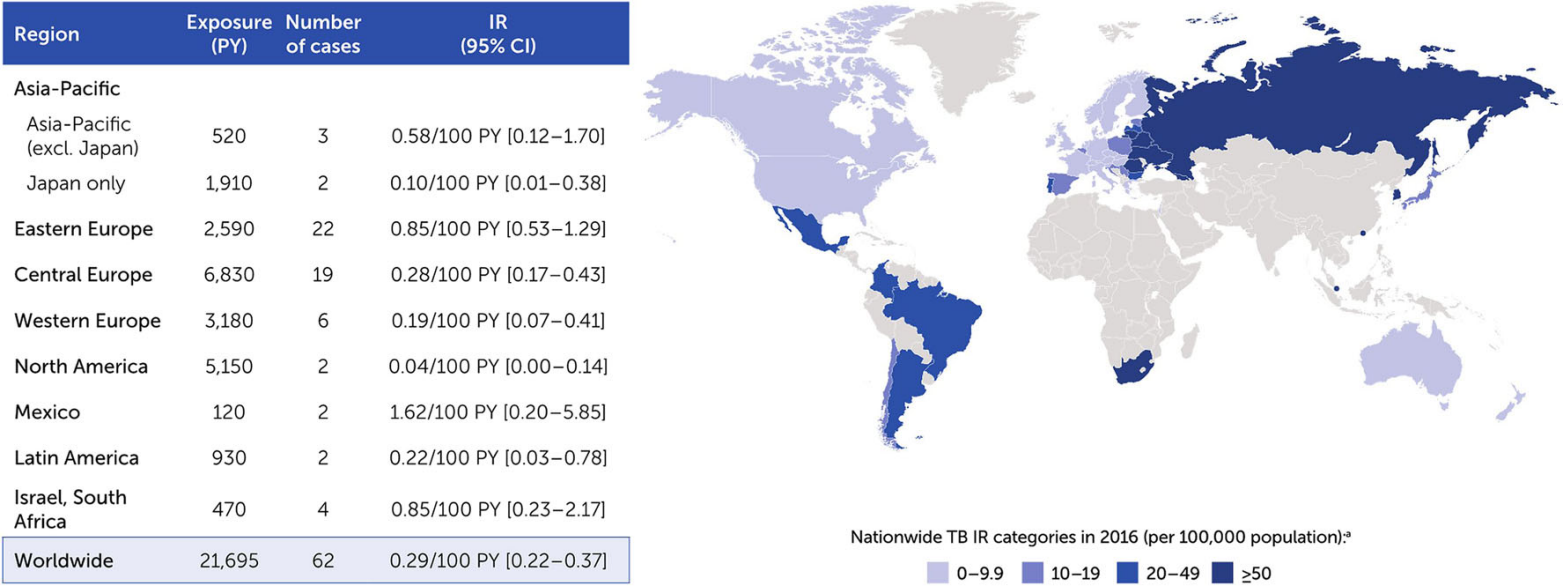
Confirmed TB cases from CZP clinical trials data by region. ^a^WHO Global Tuberculosis Report 2017; Since 2007, stricter screening rules for latent TB have been used across CZP clinical trials including a PPD cut-off of 5 mm and a TB questionnaire to help identify patients as risk. Countries shaded in blue represent those countries included in the clinical trials data set. Asia-Pacific: Australia, Hong Kong, Japan, New Zealand, Republic of Korea, Singapore; eastern Europe: Belarus, Croatia, Georgia, Romania, Russia, Serbia, Ukraine; central Europe: Bulgaria, Czech Republic, Estonia, Hungary, Latvia, Lithuania, Poland, Slovakia, Slovenia; western Europe: Austria, Belgium, Denmark, Finland, France, Germany, Greece, Ireland, Italy, The Netherlands, Norway, Portugal, Spain, Sweden, Switzerland, UK; North America: Canada, US; Latin America: Argentina, Brazil, Chile, Colombia. CZP, certolizumab pegol; IR, incidence rate; PPD, purified protein derivative; PY, patient-years; TB, tuberculosis

There were 62 expert-confirmed TB cases across clinical trials (0.29/100 PY [0.22–0.37]) (Fig. 3). The incidence of confirmed TB cases reduced after 2007, following the introduction of stricter TB screening guidelines. The pre-2007 IR of 0.42/100 PY (0.31–0.56) decreased to 0.12/100 PY (0.06–0.21) from 2007. Among all 62 expert-confirmed TB cases, 3 were reported in Asia-Pacific countries excluding Japan (0.58/100 PY [0.12–1.70])—2 in the Republic of Korea, 1 in New Zealand—with 2 additional cases in Japan (0.10/100 PY [0.01–0.38]). Across other geographic regions, the highest IR for TB were reported in Mexico (1.62/100 PY [0.20–5.85]), eastern Europe (0.85/100 PY [0.53–1.29]), and Israel and South Africa (0.85/100 PY [0.23–2.17]) (Fig. 3).

Of confirmed TB cases, 40 were classified as pulmonary and 22 as non-pulmonary/disseminated (1 in Japan and 1 in Asia-Pacific excluding Japan; 16 of the non-pulmonary/disseminated TB cases were in central [8 cases], eastern [4], and western [4] Europe). Three cases of suspected TB were classified as either doubtful or unassessable based on available pharmacovigilance information.

**Fig. 4:**
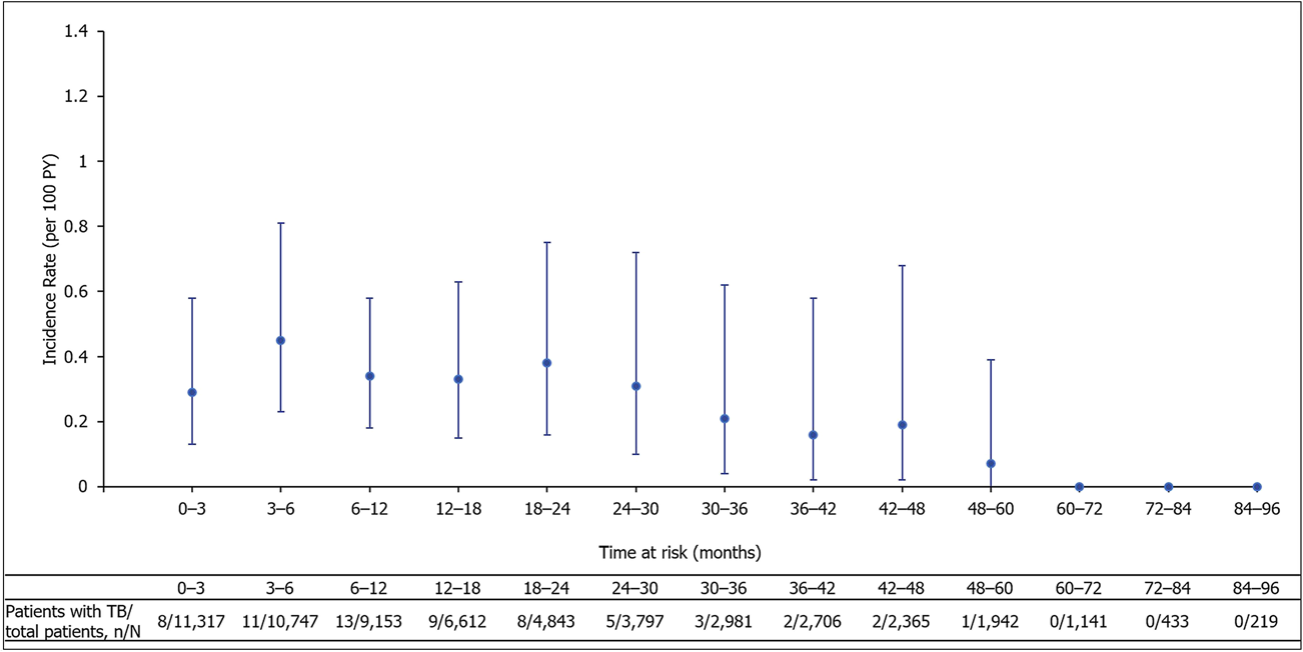
TB incidence rates over time in CZP clinical trial patients. CZP, certolizumab pegol; TB, tuberculosis. Error bars represent 95% confidence intervals

Analysis of TB cases over time since treatment initiation found the incidence of TB disease peaked within the first year of CZP treatment and decreased with longer CZP exposure (Fig. 4). For the 1141 patients exposed to CZP for more than 60 months, there were no cases of TB disease.

### Post-marketing incidence of TB

**Fig. 5:**
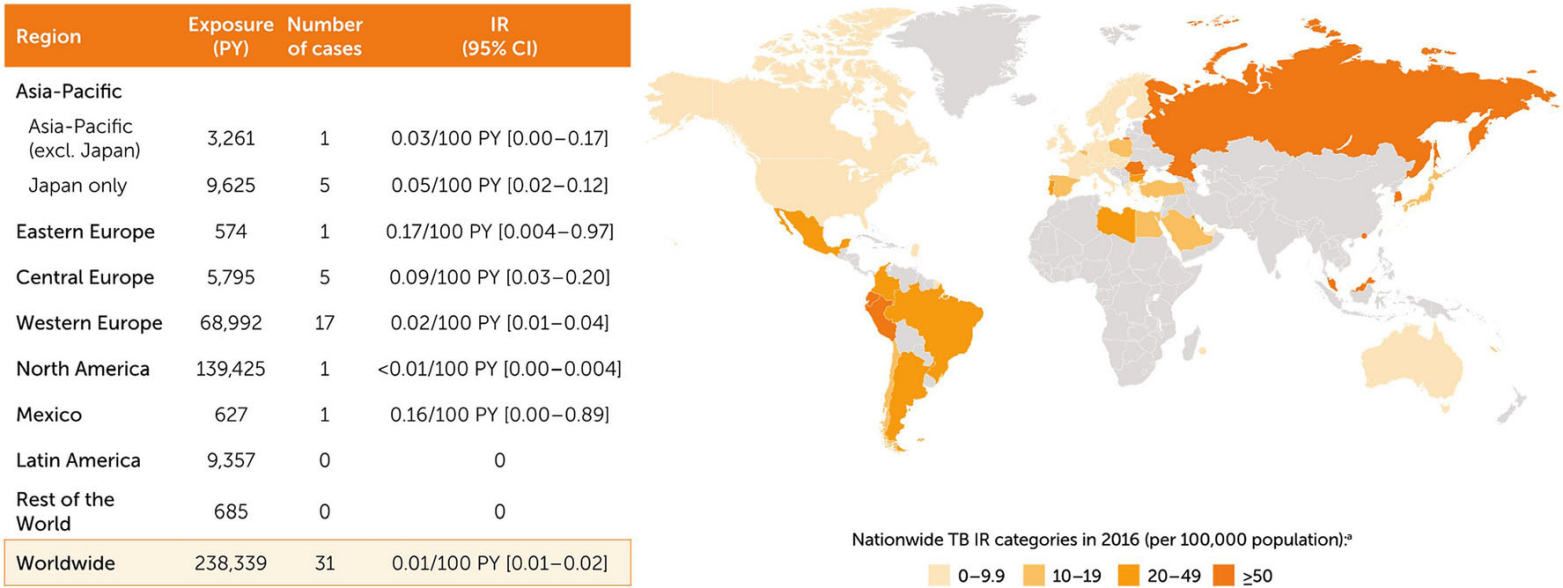
Confirmed TB cases from CZP post-marketing data by region. ^a^WHO Global Tuberculosis Report 2017; Countries shaded in orange represent those countries included in the post-marketing data set. Asia-Pacific: Australia, Hong Kong, Japan, Malaysia, New Caledonia, Republic of Korea; eastern Europe: Romania, Russia; central Europe: Bulgaria, Czech Republic, Hungary, Poland, Slovakia, Slovenia; western Europe: Andorra, Austria, Belgium, Denmark, Finland, France, Germany, Greece, Ireland, Italy, The Netherlands, Norway, Portugal, Spain, Sweden, Switzerland, UK; North America: Canada, US; Latin America: Argentina, Brazil, Chile, Colombia, Ecuador, French Guiana, Guadeloupe, Peru, Reunion; rest of the world: Bahrain, Cyprus, Egypt, Kuwait, Lebanon, Libya, Martinique, Qatar, Saudi Arabia, Turkey, UAE. CZP, certolizumab pegol; IR, incidence rate; PY, patient-years; TB, tuberculosis

A search of all AEs reported globally for post-marketing surveillance retrieved 95 suspected cases of TB in CZP-treated patients from either spontaneous sources (74 cases) or from NIS and registries (21 cases). Of these 95 cases, 31 cases were expert-confirmed (0.01/100 PY [0.01–0.02]). There was 1 case in a patient from Australia who had recently visited Thailand (included in Asia-Pacific region excluding Japan; 0.03/100 PY [0.00–0.17]), with an additional 5 cases in Japan (0.05/100 PY [0.02–0.12]) (Fig. 5), all in RA and mostly among older patients (4/5 being > 65 years old). The highest IR of TB reported across geographic regions were in eastern Europe (0.17/100 PY [0.004–0.97]), central Europe (0.09/100 PY [0.03–0.20]), and Mexico (0.16/100 PY [0.004–0.89]) (Fig. 5).

Of 31 confirmed post-marketing TB cases, 19 were classified as pulmonary and 11 as non-pulmonary/disseminated (3 in Japan and 1 in Asia-Pacific excluding Japan; the remainder of the non-pulmonary/disseminated TB cases were in western [6] and central [1] Europe). The site of infection for 1 case was unassessable based on available information. Due to the nature of post-marketing data, 50 cases of suspected TB were classified as doubtful or unassessable. There were no reports of TB disease with concomitant HIV infection.

Around half of post-marketing TB cases in CZP-treated patients occurred within the first 6 months of commencing CZP treatment (15/31 cases); the majority occurred within the first 12 months (26/31 cases) (Table 1).

**Table 1:** Time to event distribution of confirmed TB cases from CZP post-marketing data

Time to event (months)	Number of TB cases
0–< 3	5
3–< 6	10
6–< 12	11
12–< 24	2
24–< 36	1
Unassessable	2
Total	31

Based on sponsor medical review of narratives in the pharmacovigilance database

*CZP*, certolizumab pegol; *TB*, tuberculosis

### Expert-confirmed cases of HBV and HCV

Across CZP clinical trials (21,695 PY), there was 1 expert-confirmed case of HBV reactivation in a patient with RA from central Europe. No cases of HCV were reported in clinical trial patients. A search in the sponsor pharmacovigilance database, conducted to identify suspected post-marketing HBV and HCV cases, retrieved 20 suspected cases of HBV or HCV for expert review; 6 of these cases were in the Asia-Pacific region.

Of these 20 suspected cases, 5 were confirmed by expert review (0.001/100 PY [0.0004–0.003]), including 2 cases of HCV reactivation (Canada and France) and 1 case of new onset HCV in the US. There were 2 cases of HBV reactivation reported in the Asia-Pacific region (0.008/100 PY [0.001–0.028]), both in Japan. There were no confirmed cases of HCV in Asia-Pacific countries. The remaining 15 post-marketing suspected cases of HBV/HCV were classified as unassessable, doubtful, or rejected.

## Discussion

Over the past two decades, the number of patients with IMIDs exposed to biologics has increased in the Asia-Pacific region [25, 26], where nationwide rates of TB and viral hepatitis tend to be higher than in Western countries [9, 14]. However, the risk of TB and HBV/HCV in patients with IMIDs has not been well documented in the Asia-Pacific region, especially for patients receiving anti-TNF therapy (Taiwan being an exception [2, 27]). Using data pooled from CZP clinical trials across all approved indications, as well as post-marketing data captured worldwide (mostly RA and CD), we examined the incidence of TB and HBV/HCV during CZP treatment across various countries, including Asia-Pacific.

Overall, the incidence of TB disease across CZP clinical trials and post-marketing data was generally in line with nationwide rates, with more confirmed TB disease cases reported for countries or regions with a higher endemic risk of TB (Supplementary Table S3). More than 60% of confirmed TB cases consisted of pulmonary infection. This study suggests that the overall risk of TB disease during CZP clinical trials (0.29/100 PY) is in line with other anti-TNF [28, 29]. A recent meta-analysis suggested no statistically significant differences in the relative risk of TB between infliximab, adalimumab, and CZP compared with the respective placebo-controlled groups. [5] A lower incidence of TB has been reported for etanercept [30], but more recent data are lacking, especially from high TB incidence countries.

RA represented most of the total exposure in the CZP clinical trial population. Previous reports suggest that patients with RA are particularly susceptible to TB: risk is approximately two- to fourfold greater in RA compared with the general population [31], and is further increased by anti-TNF treatment [4, 20, 31]. This disease-specific risk is possibly related to the level of systemic inflammation. Furthermore, for both CZP and adalimumab, the IR of TB includes cases from RA and CD clinical trials initiated prior to 2007 when the WHO, international, and national scientific societies (including ACR, EULAR, and APLAR) introduced stricter rules for TB screening in clinical trials of biologics [14, 32, 33]. This screening program has helped to reduce the risk of TB disease during anti-TNF therapy and identify those patients at greatest risk [3]. Based on the previous safety analysis of CZP in RA clinical trials [34], implementation of these stricter screening measures resulted in a notable decrease in the IR of TB infection [3]. The results of this study further corroborate the overall reduction in the IR of TB across all approved CZP indications [18].

Real-world data on the incidence of TB in anti-TNF-treated Asian populations are still limited compared with Western countries. Studies in South Korea have reported TB IR in anti-TNF-treated populations between 0.50/100 PY and 0.34/100 PY [35, 36]. In Taiwan, the IR of TB in patients with RA was 0.68/100 PY with etanercept and 1.41/100 PY with adalimumab, compared with 0.31/100 PY in RA patients not exposed to anti-TNF [2]. In Japan, a nationwide study of 7740 patients with RA receiving adalimumab reported 22 cases of TB disease [37]. These reports highlight the importance of educating healthcare professionals and patients about effective TB screening, prophylaxis, and monitoring during anti-TNF treatment. This is especially true for patients from low incidence countries who travel to higher incidence regions, and areas where multidrug-resistant TB is prevalent.

The incidence of HBV and HCV was very low across both CZP clinical trials and pharmacovigilance data, with 3 cases of confirmed HBV and 3 cases of confirmed HCV. Although viral hepatitis is endemic in East Asian countries [10], there were no confirmed cases of HBV/HCV in Asia-Pacific countries other than Japan. However, these numbers are probably an underestimate of the true burden of HBV/HCV infection among anti-TNF-treated patients in the Asia-Pacific region, due to likely under-reporting by treating physicians.

Anti-TNF are known to be associated with HBV reactivation [8]. The highest prevalence of HBV infection worldwide is found Central and East Asian countries [10, 38]. To minimize the risk of HBV infection, screening tests and vaccination are recommended prior to biologic therapy [39]. Due to the risk of HBV reactivation in patients with chronic HBV, careful and regular monitoring and in some cases anti-viral treatment are recommended during biologic therapy [38, 40]. The implementation of nationwide immunization programs may help to reduce the background HBV burden in the Asia-Pacific region, as seen in Taiwan [41].

Anti-TNF therapy may also lead to increased HCV viremia, but research on this matter has been more limited. Evidence from low incidence countries suggests that anti-TNF have a reasonable safety profile in patients with chronic HCV infection [6, 42]. Careful follow-up of liver function and viral status are recommended [7], and the primary treatment goal in these patients should be to eradicate the HCV infection [43, 44].

This analysis represents the largest analysis of TB and HBV/HCV cases for CZP to date. Compared with the last review of TB cases in CZP-treated patients [3], this clinical trial population was enriched with additional phase 3 studies in RA, CD and PSO [18]. Overall, these data further support the consistent long-term safety profile of CZP across indications. The inclusion of post-marketing data captured worldwide, including countries where TB and HBV/HCV are endemic, contributes to a greater understanding of the safety profile of CZP in real-world settings. Furthermore, the retrospective expert review process provided a standardized, comprehensive assessment of events of potential concern, ensuring appropriate classification of TB and HBV/HCV cases and minimizing AE reporting differences across clinical specialties and geographic locations. Similar approaches have been taken for adalimumab [28] and tofacitinib [45].

However, there were limitations to this study, particularly in the context of post-marketing cases. Unlike CZP clinical trials, where patients were closely monitored with strict inclusion and exclusion criteria, the information available in post-marketing reports, including comorbidities and risk factors, was much more limited and subject to reporting bias. Therefore, the total number of TB and HBV/HCV cases in the Asia-Pacific region were likely underestimated, as post-marketing reports depend heavily on the surveillance strategy of each specific country. Additionally, reduced awareness among patients and local treating physicians of the risk of these infections during anti-TNF therapy, compared with clinical trials, may result in a higher number of cases being misdiagnosed or missed altogether. These limitations make it difficult to compare clinical trial data with post-marketing results. Finally, the data presented in this paper were not presented alongside a comparator, due to the lack of availability of comparator clinical trial and post-marketing data. However, these results are in line with those previously reported for other anti-TNF [28, 29].

It should be noted that the IR of TB in this study were compared with nationwide TB rates published in the 2017 WHO Global Tuberculosis Report [11]. However, the accuracy of these rates may vary from country to country, and between regions within countries. The global TB incidence has been falling by around 2% per year [11, 14], so IR of TB in CZP-treated populations that include cases reported in 1998, for example, may not be comparable with TB rates in 2017.

## Conclusions

In conclusion, as expected with biologic therapy and in line with WHO nationwide rates, there was a slightly higher incidence of TB in CZP-treated Asia-Pacific patients (excluding Japan) compared with CZP-treated patients worldwide. Similar results were seen for other geographic regions with a higher background incidence of TB. The post-marketing incidence of TB and HBV/HCV in Asia-Pacific countries fell within the ranges observed for patients with IMIDs treated with other biologics. A greater awareness of TB and viral hepatitis in the patient populations at risk and their treating physicians, combined with more stringent pre-biologic screening, may help to mitigate the risk of these infections, particularly in high-incidence countries.

## Electronic supplementary material

ESM 1 (DOCX 73 kb)

## Data Availability

Underlying clinical data from this manuscript may be requested by qualified researchers 6 months after product approval in the USA and/or Europe, or global development is discontinued, and 18 months after trial completion. Investigators may request access to anonymized IPD and redacted study documents which may include the following: raw datasets, analysis-ready datasets, study protocol, blank case report form, annotated case report form, statistical analysis plan, dataset specifications, and clinical study report. Prior to use of the data, proposals need to be approved by an independent review panel at www.Vivli.org and a signed data sharing agreement will need to be executed. All documents are available in English only, for a pre-specified time, typically 12 months, on a password protected portal. Data from non-interventional studies is outside of UCB’s data sharing policy.
