# Supplementary material for: Tuberculosis and viral hepatitis in patients treated with certolizumab pegol in Asia-Pacific countries and worldwide: real-world and clinical trial data
Source: Clin Rheumatol. 2020 Aug 1;40(3):867–75. doi: 10.1007/s10067-020-05248-4 (PMC7895783; doi:10.1007/s10067-020-05248-4)
Supplement: Supplementary file 1 — (DOCX 73 kb) [file 10067_2020_5248_MOESM1_ESM.docx]

Tuberculosis and Viral Hepatitis in Patients Treated with Certolizumab Pegol in Asia-Pacific Countries and Worldwide: Real-World and Clinical Trial Data

Chak Sing Lau, Yi-Hsing Chen, Keith Lim, Marc de Longueville, Catherine Arendt, Kevin Winthrop

**SUPPLEMENTARY INFORMATION**

SUPPLEMENTARY INFORMATION

Supplementary Information regarding TB Screening

Prior to 2007, for TB screening of patients considering biologic treatment the cut-off for purified protein derivative [PPD] positivity ranged from ≥5 to ≥20 mm, especially in TB-vaccinated patients. From 2007 stricter screening rules were implemented: patients who tested positive for PPD, defined as a result of ≥5 mm, received isoniazid (INH) treatment for latent TB infection (LTBI). In addition, a patient TB risk questionnaire was administered every 6 months to help identify patients at risk and to increase physician awareness of potential TB symptoms/risk factors in defined patient groups, particularly in high TB incidence countries.[^3^](#_ENREF_3) This amendment was also enforced retrospectively for all patients already receiving CZP at the time of protocol amendment.[^3^](#_ENREF_3) Some later studies incorporated an Interferon Gamma Release Assay (IGRA) test (QuantiFERON TB GOLD) as part of TB screening.

**Supplementary Table S1.** Regions and countries included in the pooled safety analysis

| **Region** | **Countries included in the CZP clinical trial analysis** | **Countries included in the post-marketing analysis** |
| --- | --- | --- |
| Asia-Pacific (excluding Japan) | Australia, Hong Kong, New Zealand, Republic of Korea, Singapore | Australia, Hong Kong, Malaysia, New Caledonia, Republic of Korea |
| Japan | Japan | Japan |
| Eastern Europe | Belarus, Croatia, Georgia, Romania, Russia, Serbia, Ukraine | Romania, Russia |
| Central Europe | Bulgaria, Czech Republic, Estonia, Hungary, Latvia, Lithuania, Poland, Slovakia, Slovenia | Bulgaria, Czech Republic, Hungary, Poland, Slovakia, Slovenia |
| Western Europe | Austria, Belgium, Denmark, Finland, France, Germany, Greece, Ireland, Italy, The Netherlands, Norway, Portugal, Spain, Sweden, Switzerland, UK | Andorra, Austria, Belgium, Denmark, Finland, France, Greece, Germany, Ireland, Italy, The Netherlands, Norway, Portugal, Spain, Sweden, Switzerland, UK |
| North America | Canada, US | Canada, US |
| Mexico | Mexico | Mexico |
| Latin America | Argentina, Brazil, Chile, Colombia | Argentina, Brazil, Chile, Colombia, Ecuador, French Guiana, Guadeloupe, Peru, Reunion |
| Rest of the World | Israel, Republic of South Africa | Bahrain, Cyprus, Egypt, Kuwait, Lebanon, Libya, Martinique, Qatar, Saudi Arabia, Turkey, UAE |

**Supplementary Table S2.** MedDRA Preferred Terms used for the selection of potential cases of TB, hepatitis B and hepatitis C

| **MedDRA Preferred Terms** | |
| --- | --- |
| **Tuberculosis** | **Hepatitis B and C** |
| Adrenal gland tuberculosis  Bone tuberculosis  Bovine tuberculosis  Congenital tuberculosis  Conjunctivitis tuberculous  Cutaneous tuberculosis  Disseminated tuberculosis  Ear tuberculosis  Epididymitis tuberculous  Extrapulmonary tuberculosis  Female genital tract tuberculosis  Immune reconstitution inflammatory syndrome associated tuberculosis  Intestinal tuberculosis  Joint tuberculosis  Lymph node tuberculosis  Male genital tract tuberculosis  Meningitis tuberculous  Oesophageal tuberculosis  Oral tuberculosis  Pericarditis tuberculous  Peritoneal tuberculosis  Prostatitis tuberculous  Pulmonary tuberculoma  Pulmonary tuberculosis  Renal tuberculosis  Salpingitis tuberculous  Silicotuberculosis  Spleen tuberculosis  Thyroid tuberculosis  Tuberculid  Tuberculoid leprosy  Tuberculoma of central nervous system  Tuberculosis  Tuberculosis bladder  Tuberculosis gastrointestinal  Tuberculosis immunisation  Tuberculosis liver  Tuberculosis of central nervous system  Tuberculosis of eye  Tuberculosis of genitourinary system  Tuberculosis of intrathoracic lymph nodes  Tuberculosis of peripheral lymph nodes  Tuberculosis ureter  Tuberculous abscess central nervous system  Tuberculous endometritis  Tuberculous laryngitis  Tuberculous pleurisy  Tuberculous tenosynovitis | Viral hepatitis carrier  Hepatitis chronic persistent  Hepatitis chronic active  Hepatitis C virus test positive  Hepatitis C virus test  Hepatitis C core antibody positive  Hepatitis C core antibody  Hepatitis C antibody  Hepatitis C antibody positive  Hepatitis C  Chronic hepatitis C  Acute hepatitis C  Hepatitis B virus test  Hepatitis B virus test positive  Hepatitis B immunization  Hepatitis B antigen  Hepatitis B antigen positive  Hepatitis B e antigen  Hepatitis B e antigen positive  Hepatitis B DNA assay  Hepatitis B DNA assay positive  Hepatitis B DNA decreased  Hepatitis B DNA increased  Hepatitis B core antigen  Hepatitis B core antigen positive  Hepatitis B core antibody  Hepatitis B core antibody positive  Hepatitis B antibody  Hepatitis B antibody abnormal  Hepatitis B antibody positive  Hepatitis B  Acute hepatitis B  Chronic hepatitis B  Congenital hepatitis B infection |

MedDRA: Medical Dictionary for Regulatory Activities; TB: tuberculosis.

Supplementary Table S3**.** Nationwide tuberculosis incidence rates in countries where CZP is approved (from the 2017 WHO Global TB Report)

| **Country** | **Population (millions)** | **TB incidence** | |
| --- | --- | --- | --- |
|  |  | **Number in thousands (95% CI)** | **Rate/100,000 population (95% CI), including HIV positive** |
| Andorra | <1 | <0.01 (<0.01–<0.01) | 6 (5.1–6.9) |
| Argentina | 44 | 11 (9.2–12.0) | 24 (21–28) |
| Australia | 24 | 1.5 (1.3–1.7) | 6.1 (5.2–7.1) |
| Austria | 9 | 0.71 (0.61–0.82) | 8.2 (7.0–9.5) |
| Bahrain | 1 | 0.18 (0.15–0.20) | 12 (11–14) |
| Belgium | 11 | 1.1 (0.97–1.3) | 10 (8.5–12) |
| Brazil | 208 | 87 (74–100) | 42 (36–48) |
| Bulgaria | 7 | 1.9 (1.5–2.4) | 27 (20–34) |
| Canada | 36 | 1.9 (1.6–2.2) | 5.2 (4.4–6.0) |
| Chile | 18 | 2.9 (2.5–3.4) | 16 (14–19) |
| Colombia | 49 | 16 (12–20) | 32 (25–41) |
| Costa Rica | 5 | 0.46 (0.35–0.58) | 9.5 (7.3–12) |
| Croatia | 4 | 0.52 (0.44–0.60) | 12 (10–14) |
| Cyprus | 1 | 0.066 (0.056–0.076) | 5.6 (4.8–6.5) |
| Czech Republic | 11 | 0.53 (0.45–0.61) | 5 (4.3–5.8) |
| Denmark | 6 | 0.35 (0.30–0.41) | 6.1 (5.3–7.1) |
| Dominican Republic | 11 | 6.4 (4.9–8.2) | 60 (46–77) |
| Ecuador | 16 | 8.2 (5.3–12) | 50 (33–72) |
| Egypt | 96 | 13 (12–15) | 14 (12–15) |
| El Salvador | 6 | 3.8 (2.9–4.8) | 60 (46–76) |
| Estonia | 1 | 0.22 (0.18–0.25) | 16 (14–19) |
| Finland | 6 | 0.26 (0.22–0.30) | 4.7 (4.0–5.4) |
| France† | 65 | 5 (4.4–5.6) | 7.7 (6.7–8.6) |
| French Guiana† | <1 | 5 (4.4–5.6) | 7.7 (6.7–8.6) |
| Germany | 82 | 6.6 (5.6–7.6) | 8.1 (6.9–9.3) |
| Greece | 11 | 0.49 (0.42–0.57) | 4.4 (3.7–5.1) |
| Guadeloupe† | <1 | 5 (4.4–5.6) | 7.7 (6.7–8.6) |
| Honduras | 9 | 3.6 (2.8–4.6) | 40 (30–50) |
| Hong Kong | 7 | 5.1 (4.3–5.9) | 69 (59–80) |
| Hungary | 10 | 0.86 (0.73–0.99) | 8.8 (7.5–10) |
| Iceland | <1 | <0.01 (<0.01–<0.01) | 2.1 (1.8–2.4) |
| Ireland | 5 | 0.34 (0.29–0.39) | 7.1 (6.1–8.2) |
| Israel | 8 | 0.29 (0.25–0.33) | 3.5 (3.0–4.1) |
| Italy | 59 | 3.7 (3.1–4.2) | 6.1 (5.3–7.1) |
| Jamaica | 3 | 0.13 (0.099–0.16) | 4.5 (3.4–5.7) |
| Japan | 128 | 20 (17–23) | 16 (14–18) |
| Kuwait | 4 | 0.96 (0.82–1.1) | 24 (20–27) |
| Latvia | 2 | 0.74 (0.63–0.85) | 37 (32–43) |
| Lebanon | 6 | 0.74 (0.64–0.86) | 12 (11–14) |
| Libya | 6 | 2.5 (1.6–3.7) | 40 (25–58) |
| Lithuania | 3 | 1.5 (1.3–1.8) | 53 (46–62) |
| Luxembourg | <1 | 0.033 (0.029–0.039) | 5.8 (5.0–6.7) |
| Malaysia | 31 | 29 (25–33) | 92 (79–107) |
| Malta | <1 | 0.058 (0.049–0.066) | 13 (11–15) |
| Martinique† | <1 | 5 (4.4–5.6) | 7.7 (6.7–8.6) |
| Mexico | 128 | 28 (21–35) | 22 (17–28) |
| The Netherlands | 17 | 1 (0.86–1.2) | 5.9 (5.1–6.9) |
| New Caledonia | <1 | 0.044 (0.037–0.050) | 16 (14–19) |
| New Zealand | 5 | 0.34 (0.29–0.39) | 7.3 (6.2–8.4) |
| Norway | 5 | 0.32 (0.27–0.37) | 6.1 (5.2–7.0) |
| Oman | 4 | 0.4 (0.34–0.46) | 9 (7.7–10) |
| Panama | 4 | 2.2 (1.7–2.8) | 55 (42–70) |
| Peru | 32 | 37 (28–47) | 117 (90–148) |
| Poland | 38 | 7.1 (6.0–8.2) | 18 (16–21) |
| Portugal | 10 | 2.1 (1.8–2.4) | 20 (17–23) |
| Qatar | 3 | 0.58 (0.50–0.67) | 23 (19–26) |
| Republic of Korea | 51 | 39 (36–42) | 77 (71–82) |
| Reunion† | <1 | 5 (4.4–5.6) | 7.7 (6.7–8.6) |
| Romania | 20 | 15 (13–17) | 74 (64–86) |
| Russia | 144 | 94 (61–135) | 66 (42–94) |
| Saudi Arabia | 32 | 3.3 (2.9–3.9) | 10 (8.9–12) |
| Serbia | 9 | 1.7 (1.4–2.0) | 19 (16–22) |
| Slovakia | 5 | 0.32 (0.28–0.37) | 5.9 (5.1–6.9) |
| Slovenia | 2 | 0.14 (0.12–0.16) | 6.5 (5.6–7.5) |
| Spain | 46 | 4.7 (4.0–5.5) | 10 (8.7–12) |
| Sweden | 10 | 0.81 (0.69–0.93) | 8.2 (7.0–9.5) |
| Switzerland | 8 | 0.66 (0.56–0.76) | 7.8 (6.7–9.1) |
| Taiwan ‡ | 24 | 11 | 46 |
| Tunisia | 11 | 4.3 (3.3–5.4) | 38 (29–48) |
| Turkey | 80 | 14 (12–16) | 18 (15–20) |
| UAE | 9 | 0.074 (0.063–0.085) | 0.79 (0.68–0.92) |
| UK | 66 | 6.5 (5.9–7.2) | 9.9 (8.9–11) |
| US | 322 | 10 (8.7–12) | 3.1 (2.7–3.6) |
| Venezuela | 32 | 10 (7.8–13) | 32 (25–41) |

†Data from France include data from 5 overseas departments (French Guiana, Guadeloupe, Martinique, Mayotte and Réunion). French incidence has been used for these overseas departments in this table. ‡Taiwan population data from National Statistics, Republic of China (Taiwan);[^63^](#_ENREF_63) TB incidence data from Lim et al. (2017).[^34^](#_ENREF_34) All other data from WHO Global Tuberculosis Report 2017.[^13^](#_ENREF_13) CI: confidence interval; HIV: Human immunodeficiency virus; TB: tuberculosis; UAE: United Arab Emirates; UK: United Kingdom; US: United States of America; WHO: World Health Organization.
